# Supplementary material for: Are carbon emissions trading and green financial instruments synergistic? -Comprehensive quantitative research based on content analysis
Source: PLoS One. 2024 Mar 7;19(3):e0298601. doi: 10.1371/journal.pone.0298601 (PMC10919601; doi:10.1371/journal.pone.0298601)
Supplement: S1 Data — (ZIP) [file pone.0298601.s001.zip › Data/地级市Z_F工作报告.zip!/║╙─╧/╞╜╢Ñ╔╜╩╨/└┤╫╘╣1⁄2╓┌║┼í╛┬φ┐╦╩2╛▌═°í┐_2012.docx]

各位代表:

现在，我代表市人民政府，向大会报告政府工作，请予审议，并请各位政协委员提出意见。

一、2011年工作回顾

2011年，是实施“十二五”规划的起步之年,也是经济形势复杂、矛盾困难较多的一年。在省委、省政府和市委的正确领导下。全市上下深入贯彻落实科学发展观，抢抓中原经济区上升为国家战略历史机遇,坚持“四个重在”实践要领,持续“学先进、比创新、看实效”,远谋近施奋力开局，较好地完成了市九届人大三次会议确定的目标任务，建设“三化”协调发展、“三地”优势突出的资源型城市可持续发展示范区迈出坚实步伐。

初步核算，全年完成生产总值1539.4亿元，增长10.8%;地方财政一般预算收入95.3亿元，增长18.3%;固定资产投资815.2亿元，增长25.1%；社会消费品零售总额410.7亿元，增长17.4%;外贸出口3.3亿美元，增长13.8%；城镇居民人均可支配收入18348元，增长13.2%；农民人均纯收入6578元，增长19.5%；城镇化率43.2%，提高1.8个百分点。

——过去的一年，我们全力抓项目、促聚集，不仅保持了经济平稳较快发展，而且推动了产业结构优化升级。重点项目建设全面加快。全市180个重点项目完成投资198亿元，提前两个月超额完成年度计划，是近三年投资时序进度最快的年份。其中，建成项目29个、新开工项目68个。荣获“全省重点项目建设管理先进单位和联审联批先进单位”。产业集聚区呈现赶超势头。10个省级产业集聚区完成投资384.6亿元，增长109.3%，占全市投资的比重提高到47%；宝丰产业集聚区进入“全省十快产业集聚区”,3个产业集聚区投资额进入全省前30位；集聚区内在建亿元以上项目占全市的50%以上，投资、项目集中度明显提高，成为县域经济新的增长极。产业结构调整取得新进展。注重从完善产业链的角度来选择新上项目，煤电、钢铁、尼龙化工、煤盐化工、高端装备制造等产业链在拉长中逐步完善贯通；培育壮大战略性新兴产业，以圣光集团为依托的医疗器械和医用耗材生产、以宝丰光伏产业园为基地的光伏产业发展迅速，行业增速在30%以上；高新区创建为国家新型工业化产业示范基地。全市规模以上工业利润171.8亿元，增长30.3%。把三产发展作为紧迫任务，文化旅游、商业地产、第三方物流等现代服务业成为三产发展的新动力，尧山-中原大佛景区创建为国家5A级景区，画眉谷、灯台架景区创建为国家4A级景区，旅游总收入增长43%。科技创新能力明显提升。新增国家级高新技术企业10家、省级工程技术研究中心3个；专利申请1150件，增长25%；煤炭综采成套装备智能系统列入国家重点发展专项，智能电网、焦炉煤气制乙炔、静脉导管三项关键技术研究列入省重大科技专项。1家企业获省长质量奖、4家企业获市长质量奖。节能减排力度不断加大。关闭小煤矿产能1330万吨、焦炭产能128万吨、钢铁产能30万吨、水泥产能160万吨，化学需氧量、二氧化硫排放量分别下降2%和4%，为发展优势产业腾出了环境容量和土地指标。

——过去的一年，我们全力抓创建、促提升,不仅完善了城镇功能，而且破解了多项城建难题。城市新区建设总体方案获批。规划面积295平方公里，功能定位“三区一中心”，即城乡一体化先行区、产业转型升级先导区、对外开放示范区、豫中南综合交通枢纽和物流中心，这对拉大城市框架、建设复合型生态宜居城市具有里程碑意义。城镇基础设施建设步伐加快。从市民最关注的问题入手，完善城市基础设施，西南环路建成通车，北环路、东环路改造升级，新建过街天桥5座，新建改建道路21条、供水供热管网140公里，城中村和旧城改造主体完工400万平方米。文化艺术中心建成投用，鹰城国际商务(商贸)中心等大型城市综合体开工建设。持续开展创建活动，成为创建省级文明城市工作先进市。六县(市)全力推动县城和小城镇提质扩容，完成基础设施投资11.7亿元，舞钢创建为国家卫生城市，宝丰创建为国家园林县城。首期20亿元的城投债券发行工作加快推进。城市管理不断加强。重拳出击整治违法占地、违法建设，拆除违章建筑155万平方米，破解了影响城市形象的老大难问题，维护了城乡规划的严肃性。大力实施城市精细化管理，启动了数字化城管建设。

——过去的一年，我们全力抓基础、促统筹，不仅发展了农业农村经济，而且彰显了新农村建设亮点。农业综合生产能力稳步提升。以水利为重点的农业基础设施建设得到加强，粮食生产战胜干旱灾害，总产达197.2万吨，连续8年丰收。农业产业化经营取得进展。新增土地流转面积11.3万亩，新建标准化养殖小区255家，新建农民专业合作经济组织283个，金牛山石榴园被评为国家级农业标准化示范区，梦想食品、源源乳业晋升为国家重点龙头企业，全市124家市级以上龙头企业年销售收入突破100亿元，带动农户58.9万户。新农村建设扎实推进。新启动新型农村社区(中心村)100个，累计启动212个，建设新民居2万多套。舞钢市新型农村社区建设为全省提供了借鉴。新建改建农村公路338公里、解决农村饮水安全人口21万人、发展沼气用户1万户、培训农民5万人、完成脱贫5.2万人。新增大面积造林26.4万亩，10个县(市、区)全部创建为省级林业生态县。南水北调工作在全省排名第一。

——过去的一年，我们全力抓开放、促改革，不仅缓解了瓶颈制约，而且增强了发展动力和活力。开放招商富有成效。引进市外合作项目440个，合同金额905.8亿元，实际到位资金525亿元，增长22%；实际利用外资3.1亿美元，增长87.7%。成功举办海峡两岸(河南)观光医疗产业合作论坛。与巴西堪布里乌市结为友好城市。要素保障机制不断创新。开展农村土地整治和城乡建设用地增减挂钩，推进存量土地集约挖潜，基本保障了发展用地。加强银企合作，扩大融资规模，全市融资总量较年初增加226.4亿元，其中，金融机构新增贷款102.8亿元，新增存贷比116.7%；新增直接融资108亿元，有力支持了经济发展。重点领域改革深入推进。以必胜的决心和有效的运作，推动煤炭企业兼并重组，关闭99家、整合保留61家，显著提高了产业集中度和安全保障能力，为煤炭工业健康发展奠定了基础。历时九年的星峰集团改制取得突破性进展，清算重组工作正有序进行。天瑞水泥在香港成功上市。普生与国药集团、华利制药与华润双鹤药业、圣光集团与华润北京医药进行战略合作或重组。医药卫生体制改革完成三年任务。文化、教育、科技、供销、集体林权等领域改革取得新进展。非公有制经济蓬勃发展。各类市场主体新增2.2万户、达到10.4万户。民间投资持续活跃，占全市投资比重达78.4%。过去的一年，我们全力抓民生、促和谐，不仅让群众得到更多实惠，而且加强了社会建设。民生工程扎实推进。把安全生产作为最大的民生，健全完善以行政首长负责制为主要内容的安全生产责任体系，创新安全监管措施，扭转了安全生产被动局面，荣获“全省安全生产责任目标考核优秀市”。持续加大民生投入,财政用于民生的支出占67.6%，“十大民生工程”全面完成。城镇新增就业12.3万人，农村劳动力转移就业7.2万人。城乡低保实现动态管理下的应保尽保，养老、医疗等社会保险实现“扩面提标”。开工建设保障性住房2.7万套，建成9890套；发放廉租住房补贴2400万元；为6000户农村贫困家庭实施了危房改造。社会事业协调发展。实施学前教育三年行动计划，新建幼儿园47所。解决城区中小学“大班额”问题有了实质性推进。完成校舍加固或重建5.4万平方米。农村广播电视村村通、农家书屋等文化惠民工程深入推进。成功主办了第二届中国曲剧艺术节、第八届中国杂技金菊奖暨第五届魔术文化节。医疗卫生设施得到加强，一批县乡基层卫生机构加快建设。成功举办了市八运会暨首届全民健身大会。人口计生工作继续保持全国先进。荣获“全国科技进步先进市”、“全国无障碍建设先进城市”、“全国残疾人工作示范城市”，被命名为“中国书法城”、“中国观音文化之乡”。民主法制和精神文明建设全面加强。坚持依法行政，自觉接受人大及其常委会监督，支持政协履行职能，认真办理人大代表建议和政协提案。全面启动“六五”法制宣传教育和依法治市工作规划。廉政建设和反腐败斗争取得新成效。扎实推进思想道德建设和精神文明创建活动，大力弘扬“三平”精神，涌现出了马俊欣、王菊红等一批先进典型。社会大局保持稳定。积极推进矛盾纠纷排查化解，信访形势向好态势得到巩固。深入推进平安建设，依法打击各类违法犯罪活动，人民群众安全感进一步提升，荣获“平安建设先进省辖市”。驻平解放军和武警消防部队在支持地方建设、维护社会稳定等方面作出重要贡献，军政军民团结更加巩固，荣获全国双拥模范城“四连冠”。外事、侨务、对台、民族、宗教、统计、史志、盐业、气象、地震、人防、档案、通信等工作都取得了新成绩。

各位代表!

过去一年的成绩来之不易。我们不能忘记关心支持平顶山的各级领导、各界朋友，不能忘记为平顶山发展打下坚实基础、作出重要贡献的老领导、老同志，不能忘记在平凡岗位上默默耕耘、无私奉献的广大干部群众。在此，我代表市人民政府，向工作在全市各条战线的广大工人、农民、知识分子、干部、驻平部队指战员、武警官兵和公安民警致以崇高的敬意!向关心、支持我市现代化建设的各界人士和国际友人、港澳台同胞、海外侨胞表示衷心的感谢!

一年来，我们在工作谋划上，注重多思考、多研究，抓大事、攻专题，把“十二五”的发展思路、战略定位分专项制定规划，并针对经济运行中的突出矛盾，制定了“破解八大难题”工作方案，用科学的规划、周密的谋划来指导实践、推动工作。在工作布局上，坚持长短结合，既立足当前抓关键，更着眼未来打基础，在基础设施建设、产业结构调整、社会事业发展、生态环境保护等方面尽可能地多做打基础、管长远的事，着力构筑“三地”优势，力促“三化”协调发展。在工作推动上，准确把握每个时期、每个阶段的主要矛盾和矛盾的主要方面,抓重点、抓关键、抓最管用最有效的事，像抓项目一样，实行网络规划、节点控制，促进了产业集聚区、招商引资、城乡建设等重点工作实现突破。在工作落实上，减少应酬，珍惜时间，潜心干事，对作出的承诺、定了的事情,盯住不放、一抓到底，敢于直面矛盾、敢于担当，干成了违章建筑拆除、西南环路建设、白龟湖整治等一批市民关注的事情。这些敢做善成、实干实效的作风应该持续。

在肯定成绩的同时，我们也清醒地看到，经济社会发展仍面临不少困难和问题：一是结构性矛盾依然突出。高新技术产业规模小、比重低，传统产业多处于产业链前端和价值链低端，服务业发展滞后，节能减排压力较大。二是投资总量依然偏低。项目谋划储备不足，发展环境不够宽松，影响发展后劲，主要指标增速在全省位次靠后。三是民生改善任务依然繁重。城乡基础设施和公益事业欠账较多，群众对就学、就医、就业、社会保障、住房保障等方面还不尽满意，物价上涨造成部分低收入群众生活比较困难。四是政府自身建设依然存在薄弱环节。一些部门服务意识差，行政效能不高；一些干部运作能力和执行能力有待提升，少数干部在岗位不在状态，不作为、乱作为，甚至存在以权谋私、消极腐败等现象。对此，我们一定高度重视，认真解决。

二、2012年工作总体要求

2012年是我市立足中原经济区建设大局，负重加压、转中求快的关键之年。总体看，今年的外部环境更为复杂困难。国际金融危机的影响短期内难以消除，外需不足可能成为常态。我国经济增速回落与物价仍处高位相互交织，需求不足成为经济运行的主要矛盾。区域竞争日趋激烈，发展压力越来越大。同时也要看到，《国务院关于支持河南省加快建设中原经济区的指导意见》明确支持我市老工业基地加快调整改造，有利于我们争取政策，建设全国重要的新型能源化工基地、现代装备研发制造基地和海内外知名的旅游目的地；我市城市新区和湛南新城规划建设全面启动，县城和中心镇、新型农村社区建设加快推进，必将释放出巨大的内需潜力；市八次党代会的蓝图谋划，提振了信心、鼓舞了士气，有利于统筹方方面面力量，合力攻坚，推动经济社会又好又快发展。

今年政府工作总体要求是：全面贯彻党的十七届三中、四中、五中、六中全会和省九次党代会、市八次党代会、市委经济工作会议精神，深入贯彻科学发展观，坚持“四个重在”实践要领，学先进、比创新、看实效，更加积极地探索和实践“三化”协调科学发展之路，更加充分地发挥“三地”优势，更大力度地引导“三个聚集”,更有实效地强化“三个根本”，把稳增长、调结构、控物价、惠民生、促改革、保稳定有机结合起来，增强忧患意识、抢抓发展机遇，转中求快、积极作为、靠正气实干，扎实推进资源型城市可持续发展示范区建设，努力保持经济社会平稳较快发展，以优异成绩迎接党的十八大胜利召开。

主要预期目标是：生产总值增长11%；公共财政预算收入增长12%；全社会固定资产投资增长22%；社会消费品零售总额增长17%；实际利用外商直接投资增长20%；外贸出口增长15%；城镇居民人均可支配收入增长10%；农民人均纯收入增长11%；城镇新增就业7.2万人；人口自然增长率控制在6.5‰以内；居民消费价格涨幅为4%左右；节能减排完成省下达任务。

实现上述要求和目标，必须立足我市实际，围绕“总目标”，把握“总基调”，着力“三个聚集”。“总目标”，就是力争我市经济社会发展主要指标增速高于全省平均水平。“总基调”，就是“转中求快”。“转”就是转变领导方式和发展方式，以领导方式转变加快发展方式转变；“快”就是提速增效，实现经济发展速度、质量和效益的同步提升。“三个聚集”，就是围绕“三化”协调发展，着力产业的聚集、人口的聚集和生产要素的聚集。

各位代表!

敬业才能成就事业，尽责才能赢得民心。完成全年目标任务，政府肩负着重要责任。无数事实证明，有了责任心，就会时刻保持危机感和紧迫感，恪尽职守，永不懈怠；就不会对竞争熟视无睹、对差距无动于衷、对落后麻木不仁。各级政府、每一个公务员都要敢于负责、能够负责、对人民负责，从现在做起，从自身做起，从细节做起，从能够做的事情做起，把各项目标任务落到实处，不断夺取经济和社会发展的新胜利！

三、2012年重点任务

今年突出抓好十个方面的工作：

(一)以新型城镇化为引领，加快城乡一体化发展。全面实施中心城市组团式发展和城市新区规划建设，逐步形成中心城区、县城、中心镇、新型农村社区(中心村)四个层级的现代城镇体系。完善城乡规划体系。高标准编制平顶山新区总体规划、中心城市组团式发展规划和综合交通、水电气暖等专项规划，完成城市近期建设规划和市域城镇体系规划，实现中心城区控制性详细规划全覆盖。抓紧完成城市规划展览馆布展并向市民开放。加快中心城区建设改造。大力推进城市新区建设，开工建设新城区金融中心、和盛广场商业综合体、移动大厦等项目，加快军分区营房、新区人民医院等项目建设，做好沿湖景观城市设计，并尽快组织实施。完成老城区旧城改造规划，鼓励企业向产业集聚区转移，启动平安大道东段、建东小区、东建材市场、华宝商场、市干休所和开源路、自由街、劳动路等区域连片开发，吸引有实力的品牌开发企业参与城中村和旧城开发改造，确保工程质量和进度。开工建设新华园怡购城，加快鹰城国际商务(商贸)中心等城市综合体建设，打造特色商业区。出台支持政策，以湛河区作为建设主体，全力推动湛南新城建设。加快建设路等城市主干道两侧、入市口和市内重要节点的绿化、亮化、美化升级改造，提升城市形象。持续搞好城市创建，完成“创卫”年度任务，确保“创森”一举成功。高水平建设数字城管系统，确保上半年建成投入试运行。加快推进中心城区与组团之间的城乡一体化发展，力争成为全省组团式发展试点城市。提高县城和中心镇建设水平。指导各县(市)调整完善城镇规划，走内涵式发展道路，提高吸纳承载能力。支持汝州、舞钢、郏县规划建设中心商务功能区，支持宝丰、叶县、鲁山规划建设特色商业区。研究出台加快中心镇建设的意见，进一步规范中心镇建设标准，以具备规模、基础较好、潜力较大的重点镇作先行试点，带动40个中心镇加快发展。大力发展特色产业集群，促进产城融合。推进新型农村社区(中心村)集聚式发展。按照“文化、生态、宜居、特色”的要求，完善、提升新型农村社区(中心村)规划。在城镇周边、产业集聚区附近、文化旅游景区以及交通便利、经济和基础条件较好的村率先启动建设，今年争取再启动建设100个。已启动建设的要扩大规模、提档升级、完善设施、引导入住，创新管理体制，加快旧址复耕。促进人口向城镇转移。落实放宽中小城市户籍限制政策，完善农民工城镇入户机制，研究制定与户籍管理相关的教育、征兵、优抚等专项配套措施，促进农村人口向城镇聚集。

(二)以新型工业化为主导，加快产业产品结构优化升级。围绕推进和引导产业聚集，大力实施工业发展腾飞工程，调优存量、扩大增量，加快发展方式转变。改造提升传统优势产业。通过加强技术改造、调整产品结构、降低生产成本，增创新优势，实现新发展。能源化工产业，依托平煤神马集团等企业，重点抓好25万吨己二酸、30万吨PVC配套25万吨烧碱等项目，努力打造国内一流的化工产品生产基地。装备制造产业，依托平高集团、平煤机、中材环保等企业，加快推进智能电网输变电设备、环保设备、高端液压支架等项目，建设在全国具有一定影响的高压开关、矿山设备、环保设备、铸件、摩托车等五大制造业基地。特种钢材产业，重点抓好舞钢公司冶金工业园、钢板深加工等项目，建设国内最大的特种钢材供应基地。新型建材产业，充分发挥煤矸石、粉煤灰资源丰富的优势，重点抓好高档陶瓷砖、高档石材、粉煤灰蒸压砖等项目，扩大产业规模形成集群效应。农产品加工业，重点抓好宝丰酒业产业升级、梦想公司3万吨饼干等项目，培育行业品牌，实现农产品加工产业基地化、标准化、集群化发展。培育发展新兴产业。依托恒基伟业、亿盛光伏和恒瑞碳化硅等企业，打造中部地区有影响的新能源产业园；依托平煤神马集团尼龙化工、海星化工、北京兴宇中科等企业，加快对位芳纶产业化进程，扩大安全气囊丝生产规模，加快建设10万吨针状焦、1亿平方米锂离子电池隔膜等项目，建设国内一流的新材料研发制造基地；依托圣光集团、巨龙公司等企业，发展高端医疗器械、色氨酸、苏氨酸等产品，建设中部地区最大的医疗器械企业孵化基地和全国重要的生物制品生产基地。同时，积极发展节能环保、物联网等项目，持续提升新兴产业比重。实施产业集聚区建设提升工程。完善支持政策，建立“增、调、降、退”动态考评调整机制，推动产业集聚区加快发展，力争全年完成固定资产投资500亿元；规模以上工业主营业务收入1100亿元，形成2个主营业务收入300亿元左右、3个超100亿元的产业集聚区；高新区创建为国家级高新区。增强自主创新能力。建立健全创新激励机制，加大科技投入力度，吸引资金、人才、技术等要素向企业聚集，组织实施好重大科技专项。全年大中型企业研发投入占主营业务收入的比例不低于3%，培育申报国家级高新技术企业6家，省级以上工程技术研究中心5个、企业技术中心4个，省级质检中心3个。继续实施质量兴市战略，争创省名牌产品5个，省长质量奖企业1家；主导或参与制定国家标准5项、行业标准2项。加大节能减排力度。完善节能减排考核体系，严格实行“一票否决”。全面落实固定资产投资项目节能评估和审查制度，实施能耗总量和单位能耗双重控制。加快淘汰“双高”行业落后产能，加强重点领域和关键环节节能技术改造，积极扶持节能、节地、节水、节材产业。持续推进公共机构节能工作。强化大气污染治理，启动大型电厂脱硝工程与考核，市区空气质量优良天数保持在300天以上。认真筹备监测PM2.5前期工作，按要求明年纳入监测范围。巩固提高白龟湖区域综合整治成果，推进昭平台水库环境综合整治，确保城市集中饮用水源地水质达标率100%。开工建设产业集聚区污水处理厂，加大水污染防治力度，出境水质综合达标率保持在80%以上。加快循环经济试点市建设，着力构建煤炭、煤化工、盐化工、有色、钢铁、电力等六大循环经济产业链。搞好经济运行调节。依法规范税收征管和非税收入管理，加强税源建设。落实结构性减税等各项税收优惠政策,进一步减轻企业和居民负担。完善铁路、公路运输协调服务机制，保障重点企业产品和重点物资运输。深入推动产销对接，引导上下游企业建立战略联盟。深化企业服务，健全要素保障机制，确保电煤等生产要素供应。加强物价调控，保持物价总水平基本稳定。大力整顿和规范市场秩序，切实维护消费者权益。

(三)以新型农业现代化为基础，加快农业发展方式转变。坚持保粮与增效并重，加大农业综合开发力度，扎实推进现代农业产业体系建设。建设高标准永久性良田。采取租赁、承包、入股等多种形式，推进土地依法规范流转。结合粮食核心区建设，进一步完善农田水利设施，继续实施“百千万”高产示范工程，确保高产创建面积稳定在110万亩以上，粮食总产争取达到200万吨。实施现代农业产业化集群培育工程。综合考虑规模、效益等因素，选择一批上下链接、合作紧密、以就地加工为主的农产品基地，集中力量培育优质农畜产品产业化龙头企业、农民专业合作社和农产品品牌，打造“全链条、全循环、高质量、高效益”现代农业产业化集群。全年新增市级以上龙头企业18家、农民专业合作社150个，新建各类标准化养殖场(区)200个。提升农畜产品质量安全水平。积极推行农业标准化生产，大力发展无公害产品、绿色食品、有机食品和地理标识产品，新增无公害农畜产品产地40个。推进重大动物疫病防控综合工程、畜产品安全可追溯工程和市场准入工程，确保农畜产品质量安全放心。改善农村生产生活条件。解决农村饮水安全人口10万人以上，新建改建农村公路300公里，改造危桥26座，新增大中型沼气工程15座、农机总动力5万千瓦。积极推进集体林权制度配套改革。加强林业生态建设，新增造林13.6万亩。做好各类实用技术培训和劳动力转移培训，新增转移就业6万人。加强扶贫开发和老区建设工作，再实现5万贫困人口稳定脱贫。落实国家强农惠农富农政策。加大财政用于“三农”的支出，确保增量和比例均有提高。继续加大农业补贴强度，认真执行重要粮食品种最低收购价，完善粮食直补政策，加大良种补贴力度，增加农机购置补贴规模。有效整合涉农资金，提高使用效益。

(四)以基础设施建设为抓手，加快提升“三化”发展支撑保障能力。谋划实施一批重点建设项目，为长远发展夯实基础。交通方面，开工建设平桐公路平顶山至舞钢段、平郏快速通道、宝丰至石龙区快速通道等项目；配合实施孟平铁路复线电气化改造、平西火车站广场改造、郏县地方铁路、焦桐高速汝州至登封段等项目；力争开工大南环、神马大道东延和武西高速尧山至栾川段等项目；积极做好尧山机场和平鲁、平叶快速通道及大北环项目前期。通过实施这些项目，提升区位交通优势，打造豫中南综合交通枢纽和物流中心。能源方面，建成平煤姚电地下输煤通道、京宝焦化焦炉煤气制液化天然气、叶县马头山风电等项目；开工建设郏县云阳寨、宝丰红石山、卫东马棚山3个风电、12座变电站、农村电网升级改造、中石油平顶山分公司宝丰成品油库等项目；加快推进姚电公司2×100万千瓦火力发电、瑞平电厂2×60万千瓦煤矸石综合利用发电、叶县天然气盐穴储备基地、蓝光电厂生物质能发电等项目前期工作。通过实施这些项目，进一步优化能源结构，提升能源保障能力和水平。水利方面，完成南水北调中线平顶山段、北汝河综合治理、24座小型水库除险加固和8个县(市、区)山洪灾害防治、2个国家小型农田水利重点县和郏县高效节水灌溉示范县、千亿斤粮食核心区、农业综合开发及中低产田改造等项目年度工程；开工建设南水北调中线配套工程及受水区城市供配水、孤石滩水库除险加固、昭平台和白龟山两处大型灌区续建配套与节水改造、白龟湖国家湿地公园、湛河水质治理等工程。加快推进“十二五”规划项目前期工作，重点抓好沙河市区段治理、市区北部和东部供水、引昭入市等项目前期工作。通过实施这些项目，提高水资源保障和防洪除涝抗旱能力。城市基础设施方面，完成新新路南段、未来大道东延、湖滨大道东延等6个项目；加快推进第二污水处理厂和污泥处理厂等项目建设；开工建设平安大道西延、开发一路北延、东平郏路市区段、平宝路改线、市区道路积水点排淤改造、山顶公园改造、湛河上游治理等16个项目；积极谋划东环路南延、姚电大道西延、科技路北延、湛北路东延等6个项目。加快实施姚电公司3、4号机组热电联产改造、城区热网建设以及天然气置换人工煤气等工程。通过实施这些项目，进一步改善城区环境，建设更加宜居、宜业、宜游的幸福家园。

(五)以提高招商实效为关键，加快构筑对外开放高地。把对外开放作为应对复杂局面和破解各种制约的全局性举措，以大开放促大发展。持续开展大招商活动。举办好第四届华合论坛，组团参加国家、省重大招商活动，有针对性地到省外、境外招商。注重产业链招商和以商招商，引导支持各类产业集聚区发挥优势，吸纳和承接国内外高层次产业转移，力争全年实际到位市外资金600亿元。进一步拓宽开放领域。加快服务业对外开放，吸引境内外投资者进入现代物流、金融、旅游、文化等领域。深化农业对外开放，重点引进食品加工、畜牧养殖与精深加工等项目。扩大基础设施和城乡建设领域对外开放，吸引境内外资金投资交通、水利、信息等基础设施，参与旧城改造、城市新区和县域城镇建设。广泛开展对外科技合作与交流。加快富士康项目引进步伐，培育一批带动能力强的龙头企业和出口基地。着力优化发展环境。认真开展“招商引资项目落地年”活动，把招商引资实效纳入目标考核体系，努力提高合同履约率、资金到位率和开工率。进一步落实各项优惠政策,切实保护投资者合法权益，真正做到项目入驻零障碍、低成本、高效率，以企业家的评价和知名企业的落户来评判我们的投资环境。

(六)以发展现代服务业为重点，加快提升第三产业水平。第三产业既是我市发展的一个短板，也是未来发展的潜力所在，必须扩大产业规模，提升发展层次。工作中突出抓好四个方面：一是旅游。抓住与省旅游局建立局市紧密合作机制的机遇，编制实施旅游目的地品牌建设和营销规划，加大旅游形象宣传和产品营销力度，推动成立大伏牛山旅游联盟，打造“中原大佛、神奇尧山、观音祖庭、近悦远来”旅游品牌。加快推进大香山风景名胜区、市游客综合服务中心等项目建设，进一步开发温泉、历史文化等资源，促进旅游与文化、农业、林业、水利、城建等融合发展，努力培育一批竞争力强的旅游企业。继续加强精品景区建设，再创建国家4A级景区2个。二是现代物流。实施物流业发展规划，加快平西、大香山、叶县和平东物流园区建设。大力发展第三方物流，引导工业企业三产分离，鼓励企业将原材料采购、运输、仓储等业务委托第三方经营，推进企业物流向物流企业转变。三是金融保险。在服务好已入驻我市的交通、兴业、广发等股份制商业银行的基础上，支持中信银行平顶山分行早日开业，为民生银行、浦发银行入驻创造条件，鼓励国有银行加大对地方企业的支持力度。稳步发展地方金融机构，继续推进农信社组建农村商业银行，新设1~2家村镇银行。争取开展保险资金投资基础设施试点。优化金融生态环境。四是商贸流通。加快传统商贸业改造升级，加强与知名商贸企业合作，优化网点布局，努力打造豫中南商贸中心。改造提升体南路等市区农贸市场，开工建设开源路新升农贸市场、东环路魏寨农贸市场，加快平西商贸中心、和谐园农贸中心建设，做好万城国际商贸广场前期工作。优化消费环境，促进居民消费。同时,加快发展商业地产、楼宇经济、总部经济等新兴业态，使之成为第三产业发展新的增长点。通过努力，培育省、市级服务业园区10个，开工超亿元重点项目14个，第三产业发展水平进一步提升。

(七)以深化文化体制改革为契机，加快文化资源大市向文化强市跨越。紧紧抓住中央深化文化体制改革、推动文化大发展大繁荣的历史机遇，以高度的文化自觉和文化自信提升文化软实力。提高全民文明素质。坚持把社会主义核心价值体系融入国民教育、精神文明建设全过程，大力弘扬“三平”精神，深入推进社会公德、职业道德、家庭美德、个人品德和青少年思想道德建设。积极开展省级文明城市和各类群众性精神文明创建，不断提高城市文明程度和市民文明素养。完善公共文化服务体系。大力实施文化惠民工程，新建629个农家书屋、500个企业职工书屋，完成广播电视“村村通”工程677个。继续实施农村(校园)公益电影放映工程，开展好舞台艺术送农民活动。完成市博物馆布展任务，确保5月1日开馆运行。做好市民活动中心项目规划。深入开展“扫黄打非”，净化社会文化环境。大力发展文化产业。深入推进宝丰文化改革发展试验区建设，加快文化创意产业园、汝瓷文化园等项目建设。推进文化与旅游融合发展，加强对尧山等自然景区的文化融入，提升知名度。围绕陶瓷、金镶玉、绢花、奇石、根雕等传统制作工艺，开发生产文化旅游产品，逐步形成规模。深化文化体制机制改革。深入推进文化企事业单位改制，支持平顶山日报社组建报业传媒集团，市文广新局组建演艺集团、文化影视传媒集团，市广电总台组建广播电视传媒集团。完成电视采编播数字化改造。积极推进县级有线电视网络整合。加强文化市场建设和管理,保护激发文化创新活力。

(八)以体制机制创新为动力，加快推进重点领域和关键环节改革。深入推进企业改革重组。依法规范星峰集团破产改制。积极推进国有物资商贸流通企业改制。基本完成市直机关所属企业脱钩改制。稳妥推进水务、热力、燃气等公用事业体制改革。加强国有资产监管。加大企业上市培育力度，加快巨龙公司、易成公司、开元特种石墨、圣光集团上市步伐。推动企业战略重组,加强与跨国公司、大型央企及国内500强企业的战略合作。加快民营经济和中小企业发展。出台进一步促进民营经济和中小企业发展的具体措施，完善项目对接、投资融资、市场开拓等服务体系。建立健全中小企业征信体系，加强融资担保机构风险防范。创新要素保障机制。吸引更多的民间资本、外来资本参与经济建设，投入实体经济。加大土地整理和违法治理力度，坚守耕地红线，实施差别化土地政策，保障重点项目和重要基础设施用地。围绕促进就业增收、满足招商引资和产业集聚区用工需求，健全人力资源开发机制，更大力度地推进和引导生产要素的聚集。

(九)以保障和改善民生为目的，加快构建和谐社会。进一步加大投入力度，继续实施十大民生工程，让群众得到更多实惠。切实加强安全生产。严格落实安全生产责任制，实行安全监管、隐患排查网格化管理，深化安全平顶山创建活动。突出抓好煤矿安全生产，严厉打击非法违法开采。加强道路交通安全专项整治，做好校车安全管理。加强非煤矿山、危险化学品、消防、烟花爆竹、建筑施工等重点行业、重点领域和商场、学校等人员密集场所的安全监管。实施重大隐患挂牌督办,坚决遏制重特大事故发生。千方百计扩大就业。实施更加积极的就业政策，稳定和扩大就业。支持小型微型企业发展，鼓励以创业带动就业。强化就业公共服务，突出抓好高校毕业生、农村转移劳动力、城镇就业困难群体、退役军人的就业问题，动态消除“零就业”家庭。不断完善社会保障体系。建立健全最低工资标准、职工工资、退休人员基本养老金、城乡低保及农村“五保”供养标准的正常调整机制。将国有、集体企业“老工伤”人员全部纳入工伤保障范围，继续增加企业退休人员基本养老金，规范完善失业、医疗、工伤、生育保险市级统筹，提高新农合住院报销比例。实现新农保和城镇居民社会养老保险制度全覆盖。落实社会救助和保障标准与物价上涨联动挂钩机制。继续办好爱心救助超市，加快推进市儿童福利院和流浪未成年人救助保护中心建设，建立完善养老服务体系。大力发展社会福利、慈善、妇女儿童和残疾人事业。着力提升住房保障水平。以公共租赁住房建设为重点，新开工各类保障性住房1.45万套,竣工1.5万套，发放廉租房租赁补贴1500万元。制定完善保障性住房建设、分配、管理、退出等制度，确保工程质量和分配公平。提升社区物业管理水平，努力打造和谐社区。认真落实国家房地产调控政策，促进房地产市场健康发展。全面发展社会事业。加大财政对教育投入，确保教育支出占公共财政支出的比例达到省定要求。加快学前教育和义务教育学校建设，全市新建、改扩建幼儿园89所，市城区新建、改扩建中小学18所。依托平顶山教育学院，积极申报幼儿师范高等专科学校。支持高校走内涵式发展道路。深化职业教育资源整合,提升办学水平。积极推进市教育园区建设。继续深化医药卫生体制改革，加强医疗卫生基础设施建设，开工建设5个乡(镇)中心卫生院、6个急救指挥中心和10个卫生监督所。大力发展全民健身事业，实施市体育村升级改造工程。加快公交场站和出租车综合服务区建设，更新公交大巴车100台、出租车300台，进一步解决群众乘车难问题。加强和创新社会管理。健全社会管理格局，畅通群众利益诉求渠道，认真排查与化解社会矛盾，信访积案化解率达到90%以上。坚持计划生育基本国策，统筹解决好人口问题，巩固全国人口计生综合改革示范市成果。深入推进“六五”普法依法治理，加强人民调解、社区矫正和法律援助工作。完善社会治安防控体系，严厉打击各种刑事犯罪活动,全力维护社会稳定。

深入开展双拥共建，支持驻平解放军、武警部队和人民防空建设。支持工会、共青团、妇联等人民团体发挥桥梁纽带作用。继续做好外事、侨务、对台、民族、宗教、统计、史志、盐业、气象、地震、档案、通信、国家安全等工作。

(十)以加强政府自身建设为保障，加快提升政府公信力和执行力。坚持用领导方式转变加快发展方式转变，不断清理观念上的“四个不善于”、方法上的“四个习惯于”和作风上的虚、俗、假、贪等不符合科学发展观要求的各种障碍，进一步提升推动力和执行力，促进行政运行更加高效、更加便民、更加廉洁。强化服务理念。围绕服务企业、服务群众、服务基层，加快政府由管理型向服务型转变。深化行政审批制度改革，以市县乡行政服务中心为主体，积极推进政务服务体系建设，最大限度地提高行政效能。加强电子政务建设，为基层和群众提供便捷服务。加大政务公开力度，对涉及群众利益和公共政策的事项办理全过程公开。积极推进财政预决算公开。加强政府公务人员教育和管理，开展争做“人民满意的公务员”活动。严格依法行政。认真依照法定权限和程序行使权力、履行职责，依法管理经济社会事务，维护法律尊严。坚决执行人大及其常委会的决议决定，自觉接受人大法律监督、工作监督和政协的民主监督，支持政协参政议政，积极办理人大代表建议和政协提案。完善行政执法程序，规范行政执法行为。做好行政复议工作，强化行政执法责任和过错追究。健全行政监督体系，加强审计监督、行政监察和行政层级监督，严格行政问责。注重实干实效。强化务实发展意识，大兴求真务实之风，说到做到、说好做好。在提高执行力上下工夫，将决策部署真正落实到具体项目、落实到责任主体，雷厉风行、锲而不舍，加强督察、务求实效。紧紧抓住事物的主要矛盾和矛盾的主要方面，以重点突破带动全局，多干管长远、惠民生的实事、好事。下决心精简会议和文件，改进会风和文风，减少迎来送往，切实把有限的时间用到谋划工作、推动落实上。坚持廉洁从政。严格落实党风廉政建设责任制，大力推进惩治和预防腐败体系建设，不断增强党的意识、政治意识、危机意识、责任意识，切实做好保持党的纯洁性各项工作。加大纠风治乱力度，认真解决损害群众利益的突出问题。严格公车管理、规范公务接待、加强因公出国(境)管理，坚决反对讲排场、比阔气、铺张浪费等奢靡之风。严肃换届纪律，树立正确导向。继续保持惩治腐败高压态势，严肃查处各类违法违纪案件，努力营造风清气正、廉洁奉公的良好氛围。

各位代表!

多少事，从来急；一万年太久，只争朝夕。我们都深感平顶山的发展慢不得，必须加快；我们都感叹时光易逝，必须珍惜。面对政府今年繁重的工作任务，我们要争分夺秒地干、夙兴夜寐地干，万众一心，奋发进取，努力创造经得起实践、人民和历史检验的业绩!

各位代表!

提前全面小康的美好前景激励着我们，500万人民的热切期待鼓舞着我们。让我们在省委、省政府和市委的正确领导下，深入贯彻落实科学发展观，正气实干，转中求快，圆满完成今年各项目标任务，以优异成绩迎接党的十八大胜利召开!
